# COVID-19 Crisis: Exploring Community of Inquiry in Online Learning for Sub-Degree Students

**DOI:** 10.3389/fpsyg.2021.679197

**Published:** 2021-07-22

**Authors:** Yui-yip Lau, Yuk Ming Tang, Ka Yin Chau, Lina Vyas, Andres Sandoval-Hernandez, Simon Wong

**Affiliations:** ^1^Division of Business and Hospitality Management, College of Professional and Continuing Education, The Hong Kong Polytechnic University, Hong Kong, China; ^2^Department of Industrial and Systems Engineering, The Hong Kong Polytechnic University, Hong Kong, China; ^3^Faculty of Business, City University of Macau, Macau, China; ^4^Department of Asian and Policy Studies, The Education University of Hong Kong, Hong Kong, China; ^5^Department of Education, University of Bath, Bath, United Kingdom; ^6^Division of Science, Engineering and Health Studies, College of Professional and Continuing Education, The Hong Kong Polytechnic University, Hong Kong, China

**Keywords:** online learning, community of inquiry, sub-degree students, network speed, gender, academic discipline, COVID-19

## Abstract

The COVID-19 pandemic has brought a tremendous impact on the pedagogy and learning experience of students in sub-degree education sector of Hong Kong. Online learning has become the “sole” solution to deal with student learning challenges during this chaotic period. In this study, we explore online learning for sub-degree students by using a community of inquiry (CoI). As such, confirmatory factor analysis (CFA) was conducted on survey data gathered from 287 sub-degree students from the business and engineering disciplines. Results indicated that the network speed for online education determines the perceived cognitive presence, social presence, and teaching presence of students, whereas gender and academic disciplines of students are not moderating factors that create a significant difference in perceived cognitive presence, social presence, and teaching presence of students. Our study findings for creating and sustaining a purposeful online learning community are highlighted.

## Introduction

In general face-to-face (also named as a traditional classroom), online, and blended (also named as a hybrid, inverted, or flipped) are the common teaching pedagogy methods in higher education institutions. The adoption of technology into teaching pedagogy has arisen in different research agendas and directions in recent years (Rasheed et al., 2020). The current advancement of digital technologies, open-source software communities, and applications provides remarkable opportunities to deliver online education. However, authors like Shea and Bidjerano (2009) consider that although the emergence and establishment of online education are well-documented, the online learning context is in need of change. This is especially true under the social conditions derived from the COVID-19 pandemic.

According to Sangrà et al. (2012, p. 152), online education is “an approach to teaching and learning, representing all or part of the educational model applied, that is based on the use of electronic media and devices as tools for improving access to training, communication and interaction and that facilitates the adoption of new ways of understanding and developing learning.” Online education is emerging as the main modality of higher education (Singh and Holt, 2013). Singh and Holt (2013, p. 98) reinforced “The Sloan Consortium found that 66% of post-secondary institutions reported an increased demand for new distance education course offerings as well as an increased demand for their existing distance education coursework.” As such, online learning is now recognized as an adaptable and portable approach for students to “possess new skills, new knowledge and new ways of learning” in a digital era (Wood and Shirazi, 2020, p. 1). Students having the opportunity to access or use an online learning system can engage in instructional materials without time and place restrictions (Lee, 2008). Also, students are able to gain a learning process from the peer assessment and question creation *via* various media styles simultaneously (Yu and Wu, 2011). In addition, students can participate in managing the content and progress of their learning (Lee, 2008). Wood and Shirazi (2020) addressed that the control of the learning environment is gradually changed from the teacher to the learner. However, the teacher is expected to provide well-structured learning materials and subject design to improve student participation and ensure relevance.

Recently, teaching supports using technologies are important for the younger generation to enhance their learning interests. Mo and Tang (2017) adopted the problem-based learning approach to facilitate students learning 3D printing technologies. Tang and Yu (2018) make use of mobile devices to deliver teaching materials to the students anytime and anywhere. Although these technologies are good to encourage learning motivation of students, the communication and interaction between peers and teachers, as well as the ability of students to confirm and reflect what they have learned, are also essential. Sub-degree students nowadays are described as “GEN Z,” “millennials,” “digital natives,” and “net generations.” As such, social networking, multimedia, and interactivity are part of the daily experiences of students. It is somehow paradoxical that the traditional way of learning and teaching in higher education comprises a lecture-style arrangement, including a massive number of students sitting in lecture theaters with minimal interaction with their teachers and peers. Such non-participating and transmission-based experiences seem to be failing to fulfill the expectations of students (Wood and Shirazi, 2020), particularly in Hong Kong, where the conditions seem to be ideal for more interactive, technology-based approaches. Hong Kong is known for having a superb infrastructure, which meets the needs of its population and contributes to the efficiency and growth of the economy. For example, according to data from the Measurement Lab (M-Lab)^1^ in 2020, it performed the second-best internet speed out of 192 countries (we will come back to this point later in this section).

Although the policies of both developing and developed countries strive toward investing in online learning systems, the behavioral intention of students and academics to use online learning systems is relatively low (Alhabeeb and Rowley, 2018). Despite this political will and the fact that the material conditions are set for online learning systems in many countries, some educators have identified key challenges from the perspectives of different stakeholders. From the perspectives of students, students may face unfolded challenges like self-regulation, technological competency and literacy, isolation, and technological complexity. From the perspective of teachers, teachers may encounter various challenges, including online learning tools, beliefs, technological operation, and technological competency and literacy. From the perspective of an institution, technology infrastructure, teacher training, and technological provision are the main challenges (Alhabeeb and Rowley, 2018; Rasheed et al., 2020). Apart from the past research studies, this study was inspired by problems related to online learning mentioned in constructive feedback given by students during formal Student Staff Consultative Group Meetings in Hong Kong higher education institutions. The researchers gained an insight into the technical problems affecting the online learning progress of students. To better understand how these technological problems affect online learning of students, the researchers considered representative academic studies, The community of inquiry (CoI) framework was identified by Garrison et al. (2000).

**Figure 1:**
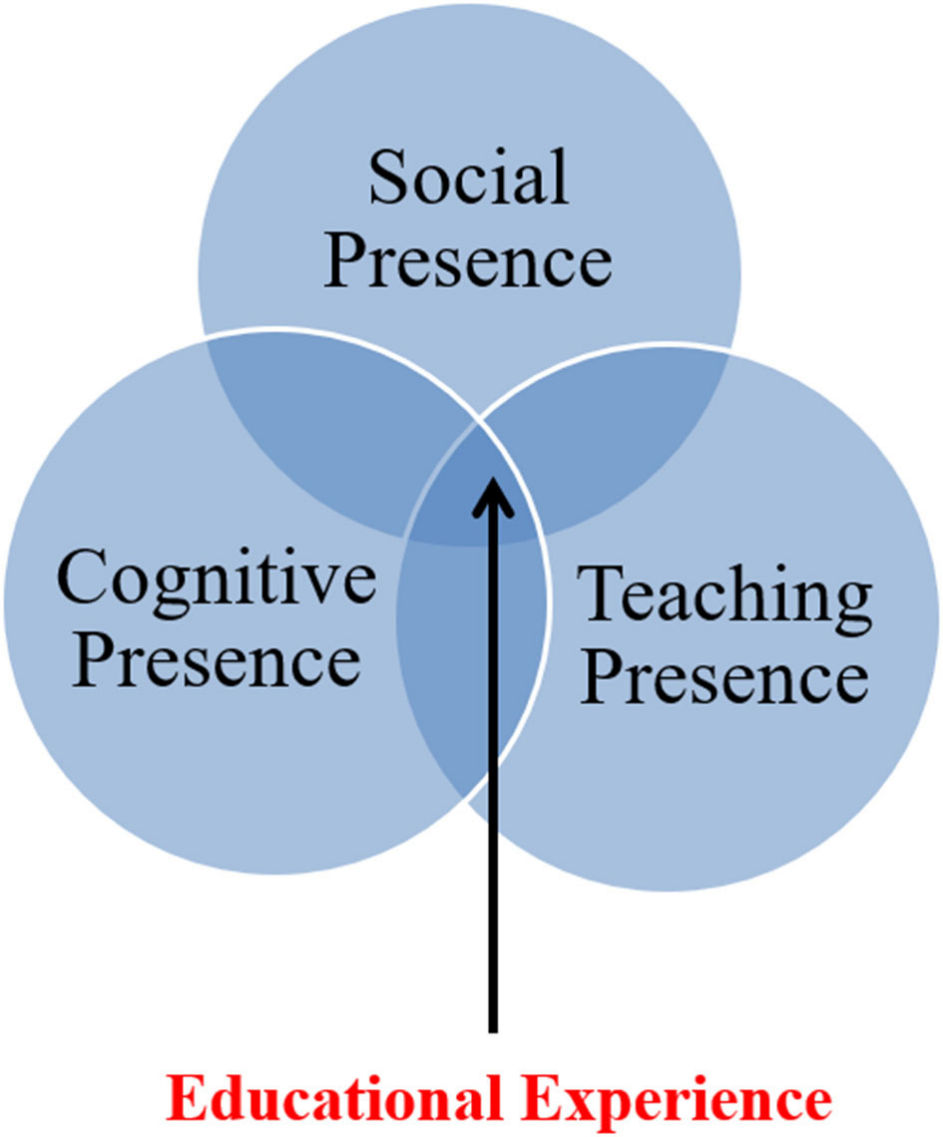
The interrelated relationship of key elements in the CoI framework. Source: Garrison et al. (2000).

The CoI framework was developed on Dewey's (1959) collaborative–constructivist learning philosophy and was later applied to online learning environments. In general, the CoI framework includes three main kinds of presence, namely, cognitive presence, social presence, and teaching presence. These three types of presence are supposed to interconnect with the CoI theoretical framework. Figure 1 illustrates the interrelated relationship of the three main kinds of presence considered in the CoI framework.

Teaching presence is described as any activity that involves teaching, such as organization and curriculum design, discourse simplification, and direct coaching to students. Social presence is defined as the activities of communicating and developing social relationships among the members of a community, such as classmates. Cognitive presence is relevant to the intellectual tasks of knowledge-building. Based on Garrison et al. (2001), cognitive presence is “*the extent to which the learners are able to construct and confirm meaning through sustained reflection and discourse*.” All these three presences are interrelated—the teaching presence is “*the design, facilitation, and direction of cognitive and social processes to realize personally meaningful and educationally worthwhile learning outcomes*” (Anderson et al., 2001).

Based on feedback collected during student–staff consultative group meetings and the CoI framework, the researchers had addressed three key research questions: (1) Is the network speed for online education associated with the perception of students of their CoI (i.e., cognitive presence, social presence, and teaching presence)? (2) Is gender of students associated with their perception of their CoI (i.e., cognitive presence, social presence, and teaching presence)? (3) Is academic discipline of students associated with their perception of their CoI (i.e., cognitive presence, social presence, and teaching presence)? The last two research questions are addressed as previous studies have found gender and academic discipline differences in technology usage behavior. For example, Venkatesh et al. (2003) theorized that gender has a moderating effect on technology usage behavior, while Park et al. (2012) found that the majority of relevance (that is, academic discipline) determines technology usage behavior. In this sense, gender and academic discipline of the students are associated with their actual usage of online learning technology. However, neither Venkatesh et al. (2003) nor Park et al. (2012) used CoI as a methodological approach. In this study, we aim at unveiling a more nuanced account of how these two factors are related to technology usage behavior through the use of a CoI approach.

To a certain extent, large-scale online learning has been forced to happen during the COVID-19 pandemic. It is a new norm that has not been explored extensively. It is argued here that what is required is a model that gives the educators a framework to understand as well as tools to overcome the challenges posed by synchronous online teaching in a particular virtual place. In this study case, the participants were sub-degree students. Specifically, these students enrolled in sub-degree programs (i.e., Associate Degree and Higher Diploma) because they attained poor examination results in their Hong Kong Diploma of Secondary Education (HKDSE). The sub-degree qualification is a significant independent exit qualification for further studies and employment in management and administrative positions at the entry level (Lau et al., 2018). Most of them were within the same age group (average 19 years of age) and had a similar experience of using online education technology. Indeed, we highlight that all the face-to-face classes were suspended in the case of higher education institutions at the outbreak of COVID-19 (also named Corona Virus) from January to May 2020. The rapid spread of COVID-19 forced various colleges and universities to close temporarily. Indeed, a normal teaching time is still questionable until we explore an effective vaccine in the world. In order to minimize the negative impacts on the learning opportunities of students, the use of online education technology was not voluntary during that time (Dhawan, 2020). For this reason, the key elements of age, the voluntariness of use, and experience were not taken into account in our study. The researchers only investigated gender and academic discipline as moderating factors. Also, the network speed issue is associated with effort expectancy—the higher the network speed for online education, the less effort an online education learner has to make. Although Hong Kong is classified as Asia World City, there is a wide wealth gap. Some sub-degree students are now living in cage-homes or bed-space apartment homes. Also, a part of sub-degree students may need to share common rooms or learning facilities in order to access to an Internet connection. Furthermore, feedback was given by students during formal Student Staff Consultative Group Meetings (see above), suggesting that the Internet speed is a common problem for sub-degree students who struggle with within Hong Kong.

The rest of the paper is divided into six main sections. The introduction is given in section Introduction; a literature review is given in section Literature Review; research methodology and results are given in sections Research Methodology and Results, respectively. Finally, the discussion of the key research findings and the main conclusion of the investigation are found in sections Discussion and Conclusion, respectively.

## Literature Review

In this section, we conduct a comprehensive literature review by exploring the terms that are relevant to our research questions. We identify theoretical descriptors (e.g., “Use of Technology,” “Community of Inquiry/CoI”); independent variable descriptors (e.g., “Gender,” “Academic Discipline,” “Male,” “Female,” “Network/Internet Speed/Efficiency,” “Students' Satisfaction Level of Network/Internet Speed/Efficiency,” “Online Education Network/Internet Speed/Efficiency”); and outcome descriptors (e.g., “Community of Inquiry/CoI Elements,” “Teaching Presence/TP,” “Social Presence/SP,” “Cognitive Presence/CP”).

In the context of online education, few research studies were addressing specific disciplines like business, education, and engineering in the CoI model (Carlon et al., 2012; Jiang and Koo, 2020). Shea and Bidjerano (2009) consider that in such particular disciplines, it is relatively complicated to incorporate technologies (e.g., wikis, blogs, streaming video) into online learning contexts in approaches that will improve student learning. Garrison and Arbaugh (2007) proposed a model for research studies to examine the generalizability of the CoI model across diverse disciplines. Later, Garrison et al. (2010a) revisited the evolution of the model, recognizing that the model still requires extra validation across disciplines and student demographics (e.g., gender and age). In response, Carlon et al. (2012) and Choy and Choon (2016) reinforced the finding that key demographics (e.g., gender and academic disciplines) affect perceptions of students of their CoI in the online learning context. Moreover, Garrison et al. (2010b, p. 32) concluded that “program of study varies according to discipline, each with unique teaching paradigms, styles of discourse and epistemologies. Given the interactive and inquiry-based focus of online communities of inquiry, different disciplines may result in unique patterns of relationships among presences. This may also be true of gender. Such differences may result in a difference in social presence, an element central to learning in an online community of inquiry.”

Our review of the literature in the field revealed that research studies investigating gender differences across disciplines in the CoI model are scarce, notably in business and engineering disciplines. Because of the known disciplinary effects in the business and engineering disciplines, these kinds of studies are urgently demanded. Also, there was a significant research gap in exploring the effect of satisfaction level of students regarding network speed for online learning, particularly framed by the CoI elements; no previous work was found that evaluated whether gender and academic discipline moderated the association between the satisfaction level of students regarding network/Internet speed for online education/learning considering the CoI elements. Furthermore, most CoI research studies (e.g., Arbaugh et al., 2010; Garrison et al., 2010a,b; Carlon et al., 2012; Arbaugh, 2013; Dempsey and Zhang, 2019; Heilporn and Lakhal, 2020) used data from undergraduate and postgraduate samples. The context of Hong Kong sub-degree students has been seriously overlooked even though it represents a large student population and is considered a unique education sector in the academic world.

Many previous studies explored the relationships (e.g., correlations and causal relationships) among the elements in the original CoI framework without involving our variables of interest (i.e., satisfaction level of students regarding network speed for online learning, along with the moderating impacts on academic discipline and gender in the proposed study). The following are some examples of these previous studies. Akyol et al. (2009) adopted mixed methods to conduct a CoI investigation, examine the transcript of online discussions, and carry out interviews to explore the main difference in the understanding of students of the CoI elements between the blended courses and online courses. Akyol et al. (2011) used mixed methods to conduct a CoI survey and analyze the transcript of online discussions to identify the difference in understanding of students of the CoI elements in long-term (13-week) and short-term (6-week) blended courses. Garrison et al. (2010b) found “teaching and social presence have a significant perceived influence on the cognitive presence and that teaching presence is perceived to influence social presence” (p. 31). Gutiérrez-Santiuste et al. (2015) used multiple regression analysis and also found that social presence and teaching presence brought an effect of the cognitive presence, while social presence has a higher effect and there is no significant collinearity between social presence and teaching presence. Rolim et al. (2019) uncovered the association between cognitive and social presence in the CoI by adopting epistemic network analysis. Some studies investigated the practice of the CoI framework, such as the studies using the Quality Matters rubric and CoI framework to guide the iterative redesign of courses to facilitate students attain learning outcomes (Swan et al., 2012, 2014), the study of combining seven fundamentals of good practice (Sorensen and Baylen, 2009) and the CoI framework to formulate online pedagogic activities for practitioners and instructors (Fiock, 2020), and the study of using computer-mediated discourse analysis on the online discussion postings to explore how the CoI elements manifested (Zhu et al., 2019).

Many other studies revised the CoI framework by integrating some new elements into the CoI framework and by examining the relationships between these new elements and the original three CoI elements. For example, Lin et al. (2015) found that self-efficacy is a complete mediator between cognitive presence and social presence. In this case, self-efficacy is a new element integrated into the CoI framework. There are other examples: Shea and Bidjerano (2010, 2012) and Shea et al. (2014) advocated the inclusion of learner presence in the CoI framework. Building on these works, Ma et al. (2017) examined the revised CoI framework and used the path analysis to find that a similar element, called learning presence, has a significant partial mediating effect in the association between the cognitive presence and the teaching presence, as well as in the association between the cognitive presence and the social presence. Furthermore, Shea and Bidjerano (2009, 2013) explored that gender, age, and academic level could generate the mediating effects of student online experience in the CoI framework.

Similar to this proposed study, some previous studies explored the relationships among the CoI elements and some new elements without revising the original CoI framework. For example, Akyol and Garrison (2008) analyzed the transcripts of online discussion postings and identified significant relationships among the CoI elements and two new elements, namely, learning motivation and expectation of students within the online program. Law et al. (2019) investigated the mediating effects of the CoI elements in the association between student recruitment and learning achievement, and between the rationale behind learning and learning achievement in a blended learning context. They found that such two new variables (i.e., student enrollment and learning motivation) influence the elements of the CoI framework, while the CoI elements influence learning performance (another new external element). In the proposed study, the new external elements are the satisfaction level of students regarding network speed for online learning and the gender and academic disciplines of students. The researchers attempted to explore the direct effect of the satisfaction level of students regarding network speed for online learning on the CoI elements and also explore the moderating effect between the satisfaction level of students regarding network speed for online learning and the CoI elements.

## Research Methodology

### Ethics

The studies involving human participants were reviewed and approved by the Hong Kong Polytechnic University. In the ethical approval process, we need to submit an ethics review checklist of human subjects (e.g., financial inducements, repetitive testing, psychological stress or anxiety, blood or tissue samples, DNA or RNA, children, unconscious patients, mentally handicapped people). Thus, our study excluded human subjects. Also, written informed consent to participate in this study was provided by the participants. The consent form addressed the information obtained from this research that may be used in future research and published.

### Data Collection

The sub-degree students participated in this study in Hong Kong during the COVID-19 outbreak in February 2020. During the study period, all face-to-face classes were completely suspended. Different learning platforms, including Zoom, Moodle, and edX, were used to facilitate online learning of students, while an online survey tool was used to facilitate data collection. The survey was open for 3 weeks after the commencement of online teaching on February 10, 2020. Six targeted academic courses in the business and engineering areas were randomly selected for the survey, and the students taking these subjects were invited to participate in the survey. The research procedure was fully explained to the participating students, and the consent of the participating students was collected when the participating students completed the online questionnaire for the survey. The students who participated in the survey study voluntarily had the right to question any part of the procedure and could withdraw from the survey at any time.

The survey questions were discussed with educators and researchers to identify appropriate content and question design. To this end, it can foster the validity of the content and make sure the correctness of the survey instruments. In particular, ambiguous wordings and double-barreled questions have been fully taken out. This process is the so-called face validity (Ngai et al., 2008). After that, we proposed our target survey respondent to conduct a pilot test of the survey. Then, we employed principal components analysis (PCA) to identify the fundamental factors that are being examined by our survey questions. Cronbach's alpha reliability values attained 0.6 as a reference point to calculate the internal consistency in our study (Nunnally, 1967). According to our analysis, the values of factor loadings were above 0.6. In other words, we identified that the reliability had obtained a satisfactory level. In the end, we arranged the ordering of the questions to create an appropriate layout of the questionnaire (Iacobucci and Churchill, 2010).

To measure the CoI presence, the online questionnaire was divided into two core parts. In the first part, student demographic information such as gender and study program was collected. The second part contained the core questions; a typical 5-point Likert scale was used to measure the level of agreement of responders with the measured statements, from strongly disagree (1) to strongly agree (5). The core part of the questionnaire consisted of 18 questions, of which five questions were used to measure the teaching presence of students, while seven and six questions were used to measure the cognitive presence and social presence of students, respectively. Table A illustrates the survey instrument used in this study. Based on the related literature (i.e., Stenbom, 2018; Gene et al., 2019; Tang et al., 2021), we construct our methodology, survey questionnaire design, and measurement scale. Through the questionnaire, we investigated whether the gender, academic discipline, and network speed of students are correlated with the perception of students about their CoI. To this end, we applied the CoI concept as the theoretical foundation to respond to our study objectives and design the questionnaire.

In this study, we distributed an online questionnaire with 400 students. About 287 respondents completed the online questionnaire with valid responses. Accordingly, the response rate was 71.75%. A total of 135 (47.04%) were male and 152 (52.96%) were female. Most were from engineering disciplines (54.01%), while 45.99% were from business disciplines. The students were participating in online learning using Microsoft Teams and Moodle. Table 1 summarizes the descriptive analysis of this study.

**Table 1:** Descriptive analysis of students who participated in the study.

**Disciplines**	**Male**	**Female**	**Frequency**	**Percentage**
Engineering	95	60	155	54.01
Business	40	92	132	45.99
Total	135	152	287	100.0

### Analytical Strategy

We first used confirmatory factor analysis (CFA) to create latent variables for the theoretical constructs postulated by the CoI model. Once we ensured the applicability of the theoretical model to our empirical data, we used a *t*-test to investigate the differences in the CoI elements by gender and academic disciplines, and ANOVA to investigate the differences in network speed.

#### Confirmatory Factor Analysis Model

The statistical analysis packages SPSS and AMOS were used to perform CFA in order to evaluate the hypothetical model at the beginning of this study (Lau et al., 2021). We adopted several commonly used models to determine the fitness acceptance levels of the CoI theoretical framework, including chi-square to degrees of freedom (χ^2^/df), comparative fit index (CFI), and standardized root mean square residual (SRMR). There is no consensus regarding an acceptable ratio for these statistics; recommendations for the level of acceptance for the χ^2^/df value of < 5 were adopted in this study (Wheaton, 1987; Schumacker and Lomax, 2004). On the other hand, we adopted the models proposed by Hair et al. (2010) and Hooper et al. (2008) for investigating the fitness of the proposed model. Recently, the SRMR enhanced the conventional RMR and provided a more meaningful interpretation of the results, while CFI was revised to form the normed fit index (NFI), which takes sample size into account and is capable of performing well even for a small sample size. The values for the CFI and SRMR range from 0 to 1.0. The CFI with values closer to 1.0 indicated a good fit, while a smaller value for the SRMR was preferred. A cutoff criterion of CFI ≥ 0.90 and SRMR < 0.05 is suggested in order to represent the close fit of the hypothetical model in the study (Bentler, 1995; Rigdon, 1996).

#### Exploring the Differences in CoI Elements

Then the mean scores and standard deviations of the 18 measured items corresponding to three CoI items were computed. The statistical difference based on gender and academic discipline differences of students was also compared. On the other hand, the effects of network speed for online learning on CoI of students in social presence, cognitive presence, and teaching presence were also investigated. Independent-samples *t*-test was used to perform the statistical test for determining the gender and academic discipline differences toward CoI, while the effect of network speed satisfaction toward CoI dimensions was compared by running ANOVA. CoI is considered an important process of inquiry and formulating scientific knowledge. It usually refers to an individual or group involved in the inquiry process in a problematic situation. Therefore, it is interesting to investigate further the difference between students studying different sub-degree programs *via* online learning. In this study, independent-samples *t*-test was also used to compare the difference between engineering and business students toward CoI in online learning. In all tests, *p* < 0.05 were adopted to identify statistical significance.

## Results

### Fitness of CoI Model

**Figure 2:**
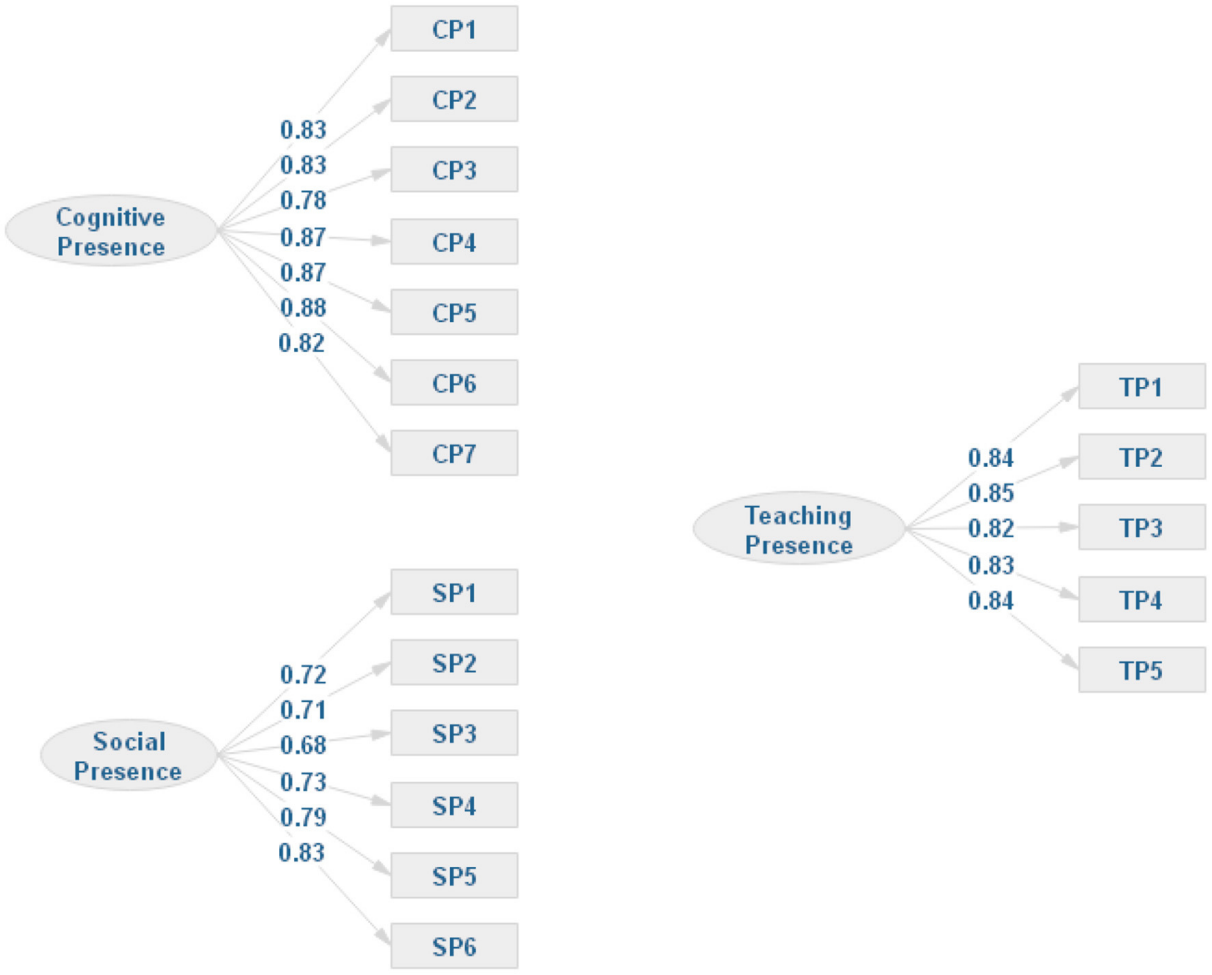
The factor loadings for each measurement item.

Confirmatory factor analysis was used to validate the hypothetical model and investigate the level of fitness of the proposed framework. The model fit tests were performed using AMOS statistical software. The fit index values of the proposed model, including χ^2^/df, CFI, and SRMR, are summarized in Table 2. In summary, it was determined that χ^2^/df = 3.856 < 5, CFI = 0.917 ≥ 0.9, and SRMR = 0.0434 < 0.05. All the models fit measurement statistics, indicating a good fit to fit the index of the target model. Factor loadings for each item were used. The values were over the value of 0.5 as proposed by Hair et al. (2010). For the cognitive presence variables, seven items were used and the factor loadings ranged between 0.78 and 0.88. The teaching presence included five items, and the factor loadings ranged between 0.82 and 0.85. There were six items included in the social presence and the factor loadings between 0.68 and 0.83. Factor loadings of all the measured items are summarized in Figure 2.

**Table 2:** The model fit test results and the target value performed using AMOS.

	**χ^**2**^**	**df**	**χ^**2**^/df**	**CFI**	**SRMR**
Target			< 5	> = 0.9	< 0.05
Results	508.985	132	3.856	0.917	0.0434

### Reliability and Validity Analyses

Statistical analyses were also performed to determine the reliability and validity of this study. The Cronbach's reliability was used, and the overall reliability is 0.936. The Cronbach's alpha for social presence, cognitive presence, and teaching presence deleted are 0.933, 0.891, and 0.894, respectively, which means the reliability was high and the items should not be removed. The correlation analysis was performed and shown that all items were significantly correlated. The correlation was between 0.804 and 0.877. The composite reliability (CR) and the average variance extracted (AVE) were used to determine the convergent validity of the analysis. The results are summarized in Table 3. The results have shown that the value of AVE for social presence was 0.4. However, according to Tang et al. (2021), as the measured CR was relatively high (>0.6), the convergent validity of the construct was still adequate.

**Table 3:** The convergent validity and correlation analysis of the measurement items.

	**Social presence**	**Teaching presence**	**CR**	**AVE**
Social presence			0.790	0.400
Teaching presence	0.809^**^		0.853	0.537
Cognitive presence	0.804^**^	0.877^**^	0.887	0.530

** *p < 0.01*.

The discriminant validity was calculated by using the heterotrait–monotrait (HTMT) ratio of the correlations (Henseler et al., 2005). The HTMT values are illustrated in Table 4. It was found that the cognitive and teaching presences pair indicated only the HTMT inference discriminant validity, while another two pairs of constructs were smaller than the HTMT0.9 criterion (Mat Yusoff et al., 2020).

**Table 4:** The heterotrait–monotrait (HTMT) ratio of the correlations.

	**Social presence**	**Teaching presence**
Teaching presence	0.899	
Cognitive presence	0.891	0.941

### Analysis of Gender Differences

In order to analyze the CoI elements of students, the mean scores were computed. It was found that the mean scores for social presence, cognitive presence, and teaching presence were 3.24, 3.43, and 3.40, respectively. On the other hand, the differences in CoI means of students were compared based on the gender of students. An independent-samples *t*-test was used for comparison. The results show that the *p*-values for the gender differences in social presence, cognitive presence, and teaching presence were 0.580, 0.513, and 0.426, respectively. Table 5 summarizes the difference between males and females in the mean scores of each CoI element. The values were higher than the level of significance (i.e., *p* > 0.05), so it is concluded that there was no significant difference in the CoI elements between males and females.

**Table 5:** The statistical analysis of each CoI measurement dimension between males and females.

**Measurement dimensions**	**Gender**		***t***	***p***
	**Male**	**Female**		
	**Mean (SD)**	**Mean (SD)**		
Social presence	3.21 (0.78)	3.26 (0.69)	−0.554	0.580
Cognitive presence	3.43 (0.79)	3.43 (0.66)	0.062	0.951
Teaching presence	3.36 (0.85)	3.44 (0.69)	−0.797	0.426

### Analysis of Engineering and Business Students

Community of inquiry refers to an individual involved in the inquiry process in a problematic situation related to online learning. There exist many different sub-degree programs available for prospective students, and it is interesting to investigate whether CoI values are different for different programs. These results can provide important insights for the development of scientific literacy in online learning. The statistical analysis was conducted using an independent-sample *t*-test. The students were categorized based on their study programs (i.e., engineering and business).

The mean scores for different measurement dimensions in CoI are summarized in Table 6. The results have revealed that in online learning, there were no significant differences in social presence for the engineering and business students (*p* = 0.716 > 0.05), cognitive presence (*p* = 0.258 > 0.05), or teaching presence (*p* = 0.091 > 0.05). The mean difference between engineering and business students was −0.032, 0.097, and 0.154 for social presence, cognitive presence, and teaching presence, respectively. A positive value means engineering students having a higher mean score than business students.

**Table 6:** The statistical analysis of each CoI measurement dimension between engineering and business students.

**Measurement dimensions**	**Division**		***t***	***p***
	**Engineering**	**Business**		
	**Mean (SD)**	**Mean (SD)**		
Social presence	3.23 (0.740)	3.26 (0.735)	−0.364	0.716
Cognitive presence	3.48 (0.703)	3.38 (0.742)	1.133	0.258
Teaching presence	3.47 (0.765)	3.32 (0.765)	1.698	0.091

### Analysis of Network Speed Satisfaction

Because network speed is one of the core elements that enhance experience of students about online learning, further analysis was therefore performed to determine the CoI elements of students for online learning based on their satisfaction levels regarding their network speed. ANOVA was used for statistical analysis. The results are summarized in Table 7. It was found that most of the students, numbering 169 (58.9%), were satisfied with network speed, 87 (30.3%) were neutral, and 31 (10.80%) were not satisfied. On the other hand, the results revealed that the mean scores for all core CoI elements were directly proportional to the network satisfaction levels. The mean score for students satisfied with network speed was significantly higher than that for students who were neutral or not satisfied with network speed at a level of *p* = 0.000 < 0.01.

**Table 7:** The effects of network speed satisfaction toward different CoI measurement dimensions.

**Measurement dimensions**	**Network speed**			***F***	***P***
	**Not satisfied**	**Neutral**	**Satisfied**		
	**Mean (SD)**	**Mean (SD)**	**Mean (SD)**		
Social presence	2.49 (0.82)	3.10 (0.54)	3.45 (0.74)	17.99	0.000^**^
Cognitive presence	2.88 (0.99)	3.21 (0.54)	3.64 (0.66)	17.10	0.000^**^
Teaching presence	2.65 (1.02)	3.21 (0.54)	3.64 (0.70)	21.12	0.000^**^

** *p < 0.01*.

## Discussion

The CoI framework has been widely adopted in online learning research for 20 years. Through the CoI framework, we aimed to investigate whether the gender, academic discipline, and network speed of gender are associated with the students; perception of their CoI, for example, as to incorporate new knowledge with their current knowledge (Hilliard and Stewart, 2019). We found a non-statistically significant difference in the CoI elements between males and females, as well as between students enrolled in business and engineering programs. In addition, we found that the network speed is crucial to strengthen the potential of students to create an in-depth and purposeful learning experience (collaborative–constructivist) (Garrison et al., 2000). In other words, it brings a considerable effect on the perceived cognitive presence, social presence, and teaching presence of students.

Gender has been recognized as a key interpreter in forecasting the learning interests and expectations of students (Moses et al., 2016). In previous studies, gender is a determining factor in affecting the academic performance of students. Traditionally, the Internet and computer culture have consorted with men (Cuadrado-García et al., 2010). In this sense, male students incline toward exhibiting superior to female students in associated computer subjects (González-Gómez et al., 2012). Authors like Ong and Lai (2006) and Lu and Chiou (2010) reinforced these findings by stating that female students felt more pessimistic about e-learning than their male counterparts. Furthermore, these authors also identified that e-learning satisfaction and value are lower among female students than among male students. Specifically, the outbreak of COVID-19 has fundamentally transformed from a traditional face-to-face classroom learning method to a new, innovative online learning method. Such an unexpected scenario has completely changed the learning environment of students, making it extremely difficult to predict how the intrinsic individual personality of students could influence their learning outcomes. It is valuable to further investigate the gender differences to inform the design of reactive strategies to support inclusive online learning during this chaotic situation.

Surprisingly, our findings indicated that gender differences are not statistically significant in the CoI elements. In other words, both male and female students had performed at comparable levels in all readiness measurements including behaviors and attitudes toward learner control, independent learning, online communication self-efficacy, the rationale behind learning, and Internet/computer self-efficacy (Hung et al., 2010). The result is similar to previous research (e.g., Kay and Knaack, 2008; Chu, 2010; Cuadrado-García et al., 2010; Hung et al., 2010). To our best knowledge, we found that our survey respondents belong to the generation known as Generation Z or Zoomers. Such generations were birthed start from 1995 (Priporas et al., 2017). Most students of Generation Z have adopted digital technology from a young age. In this sense, they are confident users of social media, innovative technological tools, and the Internet (The Washington Post, 2020). Indeed, Spender (1995) and Garrison et al. (2010a) addressed that abundant computer resources can narrow down the gender gap in online learning. Both males and females have the opportunities to master HTML and Internet protocols at the same level, or use technology with similar approaches (McSporran and Young, 2001). In addition, our female students reflected that they have not encountered the online learning environment problem described by Müller (2008) as “multiple responsibilities, insufficient interaction with faculty, technology, and coursework ranked highest as barriers to women's persistence in online environments” (Müller, 2008, p. 1). As suggested by Cuadrado-García et al. (2010), the higher education institutions design a number of well-structured exercises, provide constant teaching staff support, and integrate the team and individual activities to give fair chances for all students. Such a favorable situation could help explain why our female students score at the same levels as their male peers in the CoI instrument.

Based on Arbaugh et al. (2010) and Arbaugh (2013) studies, business and engineering disciplines are considered as “Hard” and “Applied.” Our research found that students in these two academic disciplines are no different in terms of their CoI scores. This finding suggests that the CoI framework may be appropriate for applied disciplines. According to our research findings, both business and engineering students indicated that cognitive presence, social presence, and teaching presence are important to online learning. Students are assumed to be linear philosophers who are under applied, hard disciplines; our findings propose that teaching activities will be informative and concentrated. Therefore, instructors may require to focus on fostering direct instructions. Business and engineering disciplines come to be harder in orientation; our study suggests that teachers may require performing their position as content experts for students to enrich their learning participation (Arbaugh et al., 2010). In response, Wammes et al. (2016) pointed out that mind-wandering is closely related to levels of task-based motivation. In the context of COVID-19, students could only take video classes. Students indicated that video classes make them in a low motivation for learning and increase in mind-wandering over time. Accordingly, students would take fewer notes, pay low on-task attention, and reduce memory of lecture material. Interestingly, students have a greater degree of mind-wander, and their retention of the information in the lecture is reduced. As a result, students performed poorly on an assessment (e.g., tests, examination). Teachers adopt that a naturalistic teaching approach or contextual learning is notably important (Wammes and Smilet, 2017).

Furthermore, authors like Hayes (2007), Laird et al. (2008), and Arbaugh (2013) explained that students exhibited a proactive player in generating social presence in the hard and applied fields. Based on our study, we propose that teachers may need to create chances of group cohesion and collaboration within the class. To this end, the teacher may produce learning communities of students on LinkedIn, Facebook, Twitter, and WhatsApp to enable students to maintain friendships and obtain valuable learning experiences. To a certain extent, the digital world improved collaboration and supported learning. Moreover, business and engineering disciplines are concentrated on applying latterly obtained knowledge. So, it is sensible that students expect the teacher can help them to build up new knowledge *via* online learning. Discussion forums and stimulation games online are examples of intellectual activities to generate this kind of knowledge.

The growth of network technology performs global reachability and information dissemination and permits physically segregated various students to study and communicate with each other (Kardan et al., 2013). In doing so, a larger capacity and higher-speed telecommunications systems are crucial to give the means to students to improve common communications of space over distances with different students and teachers (Ichiko et al., 2001) and facilitate pedagogical processes (Johannesen et al., 2012). In various educational research studies, network speed reflects system reliability, Internet quality, and sound technology infrastructure (Sun et al., 2008; Alhabeeb and Rowley, 2018). The higher network speed can motivate students to higher usage of online resources. Accordingly, the high-speed network environment is a “must” of an online learning system (Lee, 2008). However, Rasheed et al. (2020) provided a critical review of literature from 384 research papers. They concluded that the technological sufficiency challenges cannot be overlooked and most of the students were concerned about the possibility that low network speed could affect their online activities. Educators identified that students who have less access to a laptop or Internet access due to home location are the possibility to attain low academic achievement and unable to keep up with their classmates (Pruet et al., 2016). Osorio-Saea et al. (2021) further investigated the effect of COVID-19 on parental engagement across 23 countries. Their home location determines the engagement, acceptance, and confidence of students toward the online learning environment and culture. To our best knowledge, students live in a rural area where there is a poor technological infrastructure or share a single device with siblings. Such an unfavorable situation generates a barrier for students to full engagement in remote learning and connectivity during the pandemic.

Clearly, the growing speed of data transfer and connectivity generates higher opportunities for interfacing within the learning and teaching spaces. Extensive connectivity *via* Bluetooth, infrared, and WiFi fosters the adoption of mobile devices like laptops, mobile phones, and tablets (Wood and Shirazi, 2020). If adequate network speeds and advanced mobile devices are available, teachers can respond and support discussions that explicate ideas and encourage learning. In this way, network speed fostering teaching presence produces student-centric and energetic learning environments where teachers and students are an equal technological playing field in the learning experience. To a certain extent, network speed encourages teachers to set up learning tasks, timetables, and module content, to monitor and manage intentional reflection and teamwork, and to make sure that students attain the intended learning outcomes by giving real-time information and clear orientation and determining needs (Garrison et al., 2010a).

Currently, higher education institutions are inclined toward establishing student-centric learning environments. Such student-centric learning environments are created by social presence. This generates “a climate that supports and encourages probing questions, skepticism, and the contribution of explanatory ideas” (Garrison, 2017, p. 37). Optimal network speed is expected to optimize learning impact on peer assessment and equip teachers to give constructive and prompt responses about the work of each student. Indeed, high network speed supports important pedagogical characteristics (e.g., online submission, automatic notification, asynchronous and synchronous interaction, online discussion forum, and various kinds of instant feedback, to name but a few; Yu and Wu, 2011). Wood and Shirazi (2020) stated that the majority of students expressed that network speed and different communication formats foster discussion with their classmates. Nevertheless, network speed facilitates social presence to perform group cohesion, open communication, and effective communication. Students who perform a high level of social presence can increase their involvement and participation in the learning environment and collaborate with their classmates to strive toward a common goal (Hilliard and Stewart, 2019).

Under CoI, cognitive presence indicates the inquiry and learning process, including recognizing a task/problem, combining ideas, and analyzing possible solutions. In principle, cognitive presence is anticipated and formed by teaching and social presences (Garrison et al., 2010b). In the research study, we explored that the network speed for online education associated with the perception of students for all key CoI elements. In this sense, the network speed enables students to participate in knowledge creation and critical thinking through sustained communication and reflection. Network speed supports a higher level of learning. In turn, a higher level of learning is a comprehensive multi-based exercise that is connected with an inspiring incident, examination, amalgamation, and intention (Kanuka and Garrison, 2004). Eventually, it can foster the transformation of “metacognitive awareness essential to worthwhile and continued learning” (Garrison, 2017, p. 52). In the online learning process, metacognitive awareness fosters modification backed by the response. It is students have access to instant communication with teachers, facilitated by the network speed (Hilliard and Stewart, 2019). In addition, the network speed can help students minimize interruptions when setting learning goals and enable their learning progress to go smoothly. As a result, students tended to achieve considerable improvement in their learning outcomes when they had metacognitive awareness (Azevedo and Cromley, 2004).

## Conclusion

With the effect of COVID-19, the traditional face-to-face teaching approach has been completely replaced by an innovative online teaching pedagogy. Nevertheless, different stakeholders (e.g., teachers, students, and higher education institutions) are under a transition period in a response to the online learning environment. In this context, our study focuses on Hong Kong sub-degree students. We consider that it is important to focus on this modality of education because, as evidenced by the literature, sub-degree students have been seriously overlooked in the past two decades, and because the sub-degree student sector represents a large proportion of the higher education sector in Hong Kong. In our research study, we adopt the CoI framework to explore the association between its three interrelated elements (i.e., cognitive presence, social presence, and teaching presence) and program for students about the study, gender, and satisfaction level of network speed.

Our results show that gender has no significant association with none of the CoI elements: cognitive presence, social presence, and teaching presence. We claim that one of the reasons for this somewhat surprising finding is that members of Generation Z have more positive attitudes toward innovative technological tools, the Internet, and social media. They are, therefore, more comfortable and better prepared suddenly imposed online learning environment (regardless of their gender). Clearly, it is easier for teachers to design and implement courses and to attain learning outcomes when students are more engaged in communication and collaboration during the learning process. In other words, it is easier when students are more capable of knowledge construction and critical thinking (Hilliard and Stewart, 2019). Our findings also suggest that there exists no statistically remarkable distinction neither in cognitive presence, in social presence, nor in teaching presence between students in business and engineering disciplines. The rationale behind this, we claim, is that both disciplines are classified as hard and applied. Previous research has found that as disciplines moved closer to hard and applied approaches, online learning generates a stronger association with the CoI framework (Arbaugh, 2013).

The students who participated in our study declared to be concerned with their online learning in connection with the network speed. As expected, the satisfaction level of students about the network speed for online education has a positive effect on the cognitive presence, social presence, and teaching presence. In practice, the network speed is determined by different factors, for instance, technological tools, online learning software capabilities, study locations, to name but a few. Indeed, the slow network speed induces an interruption of the teaching process, poor online learning experience, and student isolation.

Some limitations for consideration in future research have been explored in our research. Nevertheless, this research study generates a foundation work for future research. First, this research study only focused on the “Hard & Applied” discipline. Further research could include projects that compare students from different disciplines like “Hard & Applied” and “Soft & Pure.” This could contribute to generalize our research findings and deepen our awareness of the associations between the cognitive presence, social presence, and teaching presence of students and their disciplines. Second, our research study only considered online learning pedagogy. Accordingly, a future study could examine different learning pedagogies, for example, conventional face-to-face teaching, blended learning, and innovative online learning modes within the same group of students. This type of project can provide useful guidance and constructive advice to teachers and higher education institutions to design and implement appropriate learning pedagogies for different types of students accordingly, particularly in chaotic times. Third, self-reported data were employed that may be depending on the report of survey respondents accurately and willingness to answer. Students may be not willing to report actual behavior because of insufficient knowledge and possibly personal repercussions. Finally, the data were gathered mainly from students. In the future, we may collect data from numerous stakeholders like government bodies, policymakers, educators, and service providers (i.e., higher education institutions) through focus group discussions to obtain wider perspectives and create thorough data for analysis. As such, mixed research approaches, including quantitative and qualitative, could counteract the pitfalls of a purely quantitative or qualitative research approach.

## Data Availability Statement

The raw data supporting the conclusions of this article will be made available by the authors, without undue reservation.

## Ethics Statement

The studies involving human participants were reviewed and approved by the Hong Kong Polytechnic University. Written informed consent to participate in this study was provided by the participants.

## Author Contributions

Y-yL contributed to the first draft of the article, conception, and design of the study. YT contributed to the first draft of the article, performed the statistical analysis, and organized the database. KC secured the research funding and data collection. LV and AS-H proof read the article and data collection. SW performed data collection. All authors contributed to manuscript revision, read, and approved the submitted version.

## Conflict of Interest

The authors declare that the research was conducted in the absence of any commercial or financial relationships that could be construed as a potential conflict of interest.

## Footnotes

^1^M-Lab, a nonprofit consortium of researchers and corporations, tasked with documenting the quality of the Internet. More information can be found at: https://www.measurementlab.net/.

## Appendix A

**Table A:** The questions used to measure the CoI in the survey.

**Items**	**Questions**
SP1	I have the chances to express my opinions
SP2	I can interact with fellow students formally after classes
SP3	I can interact with fellow students informally during classes
SP4	I have enough collaborative activities
SP5	I enjoy participating in the course activities
SP6	I have a sense of belonging to the course
CP1	I can easily acquire knowledge from the course
CP2	I can identify problems encountered by the subject
CP3	I can explore more information related to the course from other means of learning (e.g., videos, games, and discussion)
CP4	I can link what I have learned from the course
CP5	I can reflect on what I learned
CP6	The course enables me to explore more ideas and integrate ideas into solutions
CP7	The course equips me to have higher thinking skills
TP1	The guidelines provided are clear on learning
TP2	The tasks distributed are moderate learning
TP3	The course structure is innovative
TP4	The teaching and learning tools facilitate our learning
TP5	The information delivery is satisfaction
